# The apparent surface free energy of rare earth oxides is governed by hydrocarbon adsorption

**DOI:** 10.1016/j.isci.2021.103691

**Published:** 2021-12-25

**Authors:** Junho Oh, Daniel Orejon, Wooyoung Park, Hyeongyun Cha, Soumyadip Sett, Yukihiro Yokoyama, Vincent Thoreton, Yasuyuki Takata, Nenad Miljkovic

**Affiliations:** 1 Department of Mechanical Science and Engineering, University of Illinois at Urbana–Champaign, Urbana, IL 61801, USA; 2 Department of Mechanical Engineering, BK21 FOUR ERICA-ACE Center, Hanyang University, Ansan, Gyeonggi 15588, Republic of Korea; 3 International Institute for Carbon Neutral Energy Research (WPI-I2CNER), Kyushu University, 744 Moto-oka, Nishi-ku, Fukuoka 819-0395, Japan; 4 Institute for Multiscale Thermofluids, School of Engineering, University of Edinburgh, Edinburgh, EH9 3FD, UK; 5 Department of Electrical and Computer Engineering, University of Illinois at Urbana–Champaign, Urbana, IL 61801, USA; 6 Materials Research Laboratory, University of Illinois at Urbana–Champaign, Urbana, IL 61801, USA; 7 Department of Materials Science and Engineering, Norwegian University of Science and Technology (NTNU), 7491 Trondheim, Norway

**Keywords:** Inorganic materials, Materials science, Materials chemistry, Materials characterization, Materials application

## Abstract

The surface free energy of rare earth oxides (REOs) has been debated during the last decade, with some reporting REOs to be intrinsically hydrophilic and others reporting hydrophobic. Here, we investigate the wettability and surface chemistry of pristine and smooth REO surfaces, conclusively showing that hydrophobicity stems from wettability transition due to volatile organic compound adsorption. We show that, for indoor ambient atmospheres and well-controlled saturated hydrocarbon atmospheres, the apparent advancing and receding contact angles of water increase with exposure time. We examined the surfaces comprehensively with multiple surface analysis techniques to confirm hydrocarbon adsorption and correlate it to wettability transition mechanisms. We demonstrate that both physisorption and chemisorption occur on the surface, with chemisorbed hydrocarbons promoting further physisorption due to their high affinity with similar hydrocarbon molecules. This study offers a better understanding of the intrinsic wettability of REOs and provides design guidelines for REO-based durable hydrophobic coatings.

## Highlights

• REOs are intrinsically hydrophilic but become hydrophobic as they adsorb hydrocarbons
• Our results demonstrate that both physisorption and chemisorption occur on the surface
• The adsorption of hydrocarbons was confirmed by multiple surface chemistry analysis
• Our work offers a better fundamental understanding of the intrinsic wettability of REO

## Graphical abstract

**Figure:**
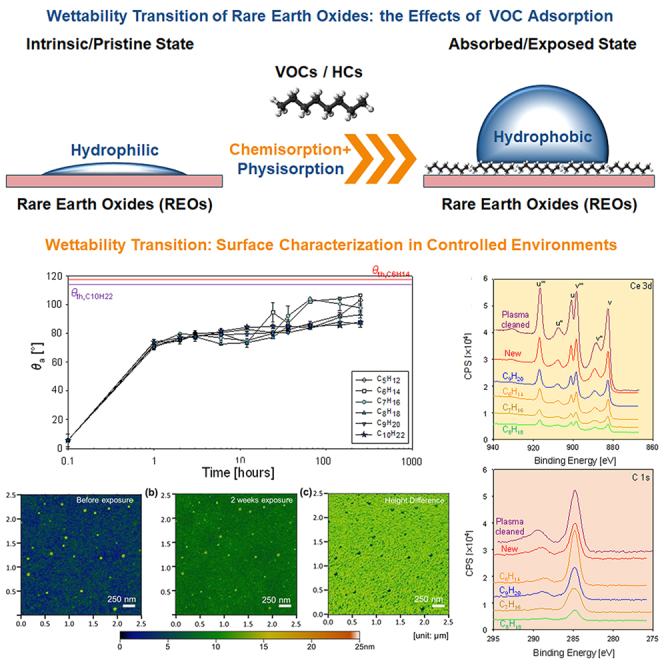

## Introduction

Scientific knowledge is driven by the advancement of our understanding of basic science and by debate generated from competing hypotheses. In the last decade, the wettability of rare earth oxides (REOs) has been a topic of argument in the scientific community. REOs were initially reported to be intrinsically hydrophobic (Azimi et al., 2013), with follow-up studies demonstrating REOs to be intrinsically hydrophilic with a slow transition to hydrophobicity upon exposure to the ambient environment (Lundy et al., 2017; Preston et al., 2014). Interestingly, similar debates raged over half a century ago regarding the intrinsic wettability of noble metals and whether they are intrinsically hydrophilic (Gardner and Woods, 1977; Smith, 1980) or hydrophobic (Erb, 1973). More recently, similar competing hypotheses have been deliberated regarding the wettability of graphene (Li et al., 2013; Munz et al., 2015) and other two-dimensional materials (Kozbial et al., 2015).

For the past six decades, scientists have reported conflicting results regarding gold and its intrinsic hydrophobicity (Erb, 1973) or hydrophilicity (Gardner and Woods, 1977; Smith, 1980). Although theoretical and experimental studies supported the idea that gold was hydrophobic, experimental studies in controlled conditions such as ultra-high vacuum (<10^−6^ Pa pressure) confirmed the hydrophilic nature of gold. Wettability measurements and surface chemistry analyses concluded that noble metals such as gold and platinum are inherently hydrophilic but they exhibit “acquired” hydrophobicity owing to contaminants adsorbed from the environment (Bernett and Zisman, 1970; Bewig and Zisman, 1965; Gardner and Woods, 1977). Contaminants from the atmosphere generally consist of non-polar hydrocarbon organic materials referred to as volatile organic compounds (VOCs). Therefore, VOC adsorption on a free surface can mask the intrinsic wettability of substrates, lowering the surface free energy at the interface with the consequent increase in non-wetting behavior. VOCs are ubiquitous and present in our atmosphere in parts per million (ppm) or parts per billion (ppb) concentrations depending on the location and chemical species of interest. These VOCs arise from livestock, plant life (Guenther et al., 1995), and man-made practices (Gligorovski and Abbatt, 2018; Valsaraj, 1994). Nonetheless, the identification and quantification of these VOCs and, more importantly, their effects on the wettability of solid surfaces remain poorly understood.

The designation of REOs originates from the scarcity and economically limited availability of rare earth elements (REEs), which mainly refer to lanthanides (atomic number from 57 to 71) along with scandium (Sc) and yttrium (Y). The REEs have been widely utilized in the past as catalysts (Goonan, 2011; Hussein, 1996; Sattler et al., 2014; Trovarelli et al., 1999), magnetic materials (Goonan, 2011; Sugimoto, 2011), and electronic components (Goonan, 2011; Kakushima et al., 2007). Hence, the precise characterization of REEs wettability is paramount for the design of these industrial processes and thermal- or energy-related applications. Rigorous wettability characterization of REOs was first reported in 2013, showing intrinsic hydrophobicity (Azimi et al., 2013). The study suggested REO hydrophobicity stems from their unique electronic configuration having octet outer-shell-filled 5s^2^p^6^ orbitals with empty orbitals on the 4f inner shell, resulting in a lack of surface polarity. However, follow-on work proposed an opposite hypothesis, which pointed to REO intrinsic hydrophilicity with acquired hydrophobicity due to airborne VOC adsorption (Preston et al., 2014). Follow-on studies have demonstrated the acquisition of hydrophobicity through transient contact angle measurements and X-ray photoelectron spectroscopy (XPS) on holmium oxide (Ho_2_O_3_) and cerium oxide (CeO_2_) exposed to laboratory environments. Rigorous experiments showed a contact angle increase with time that was correlated to the increase in carbon content on the outermost surface of the REOs as VOCs and hydrocarbons adsorb from the air, eventually changing the physicochemical properties of the surface (Preston et al., 2014). Thereafter, a second report confirming the initial results and contradicting the VOC mechanism emerged (Khan et al., 2015). This follow-on work attributed the increase in contact angle on CeO_2_ surfaces due to the transiently decreasing O/Ce ratio, ruling out the relevance of carbon content on surface wettability as surface carbon content remained the same in ultra-high-vacuum (UHV) conditions.

Given the lack of consensus on mechanisms governing REO wettability, a revived interest has developed aimed at understanding how the wettability of solid surfaces exposed to the atmosphere is governed by the physicochemical and electronic properties of the free surface for REOs (Lundy et al., 2017), metals, oxides (Yan et al., 2019, 2020), and non-metals (Li et al., 2013). In addition to experimental approaches, others have attempted to find evidence to uncover the mechanisms governing wettability using computational methods such as molecular dynamic (MD) simulations. A past study investigated the effect of polarity, i.e., oxide and hydroxide termination of the outermost surface, interfacial hydration structure, and topography, on wettability using MD simulations. They found that the orientation of the water molecules depended on the polarity of the surface termination (Giovambattista et al., 2007, 2009). Follow-on work using density functional theory (DFT) was used to explain the intrinsic hydrophobicity of REOs (CeO_2_ and Nd_2_O_3_) and went as far as to propose a mechanism associated with dissociation of interfacial water and oxide reduction (Carchini et al., 2016). The DFT simulations showed that surfaces terminated with hydroxyl groups formed by lattice oxygen (O_lat_H) can govern the shift in surface wettability from hydrophobic to hydrophilic. Others have utilized DFT to show that oxygen terminated CeO_2_ surfaces (1 0 0) are unstable (Fronzi et al., 2019). Thus, the surface tends to be dominated by the lower index configuration (1 1 1), which shows the same results reported with the previous experimental studies (Tam et al., 2018). The simulation results suggest that the wettability of CeO_2_ may vary from hydrophilic to hydrophobic owing to surface atomic orientation.

Even more recently, several important experimental studies have been published supporting the idea that adsorption of non-polar VOCs is responsible for the hydrophobicity of REOs. The presence of VOC adsorption masks the intrinsic wettability and/or the oxidation state and unique electron configurations of REOs (Külah et al., 2017; Lundy et al., 2017; Preston et al., 2014), boron nitride nanotubes (Boinovich et al., 2012), and other metallic surfaces (Chang et al., 2010; Orejon et al., 2019; Shirazy et al., 2012; Yan et al., 2019). A seminal experimental study reported the contact angle increase on REOs and RE-nitrides as a consequence of VOC adsorption from the environment (Külah et al., 2017). The results suggested that the hydrophobicity of gadolinium (III) oxide (Gd_2_O_3_) and CeO_2_ was caused by the promotion of a tetracene layer that is 2 nm thick as a consequence of the VOCs adsorption. In parallel, a second independent experimental study using controlled ambient conditions under nonane (C_9_H_20_) and perfluorononane (C_9_F_20_) ambient showed that the transition of wettability is not affected by the different oxidation states of CeO_2_ (Ce(III) and Ce(IV)), originating instead from the adsorption of the hydrocarbons as demonstrated by XPS (Lundy et al., 2017).

Inspired by the continuing debate, many researchers have focused on developing methods to fabricate durable non-wetting coatings using REO materials. Atomic layer deposition of thin (∼50 nm) films of Er_2_O_3_, Dy_2_O_3_, La_2_O_3_, CeO_2_, and Y_2_O_3_ on polished Si (1 0 0) wafers and Si nanowires (Oh et al., 2015) was used to develop manufacturing knowledge for the fabrication of thermally stable REO coatings based on the assumption of intrinsic hydrophobicity. More recently, the development of superhydrophobic coatings using solution precursor plasma spray with Yb_2_O_3_ has been demonstrated (Xu et al., 2018a, b and c). Although manufacturing and fabrication knowhow has increased, the governing mechanism of REO wettability remains in question.

Here, we thoroughly investigate the fundamental mechanisms governing the wettability of REOs in an attempt to test the acquisition hypothesis. We focus on rigorous physical and chemical characterization of the surfaces of interest, with a focus on wettability (contact angle) and surface chemistry (XPS, time-of-flight secondary ion mass spectroscopy [ToF-SIMS], and atomic force microscopy [AFM]). We show that chemisorption of hydrocarbons occurs on REO surfaces, which further promotes physisorption. We correlate the surface chemistry to wettability transition to conclusively demonstrate the hydrophobicity acquisition mechanism stemming from VOCs.

## Results

### Wettability transition

To elucidate the mechanism governing the wettability of REOs, we first established an experimental sequence to enable an REO surface to adsorb hydrocarbons and VOC from air and systematically study the wettability and surface chemistry. First, we measured the contact angle of the sputter target samples immediately after breakage (within 1 min) of the Ar seal to minimize adsorption of hydrocarbons and VOCs. All samples were received from the manufacturer sealed in Ar packaging. Once opened and characterized for contact angle, the test samples were left exposed to the indoor laboratory environment at the University of Illinois in Urbana, Illinois, United States (40°06′N, 88°13′W).

We also measured the apparent contact angle on the different surfaces immediately after (within 1 min) oxygen (O_2_) plasma and organic solvent cleaning, as well as after fixed time intervals up to 600 h. We chose CeO_2_, Er_2_O_3_, and Yb_2_O_3_ as the REO materials; Cu as a transition metal; Nb_2_O_5_ as a transition metal oxide; and SiO_2_ as a control sample. Table 1 summarizes the apparent advancing and receding contact angles measured on the samples. The initially measured apparent advancing contact angle on Cu and CeO_2_ was ≈120° and ≈110°, respectively, indicating that the surfaces had already adsorbed hydrocarbons or contaminants prior to Ar sealing. For Er_2_O_3_, the contact angle was not measurable (0°) owing to porosity of the sputter target stemming from the sintering manufacturing process. Deposited water droplets completely wicked within the porous structure of the target immediately after cleaning and for any exposure time thereafter. We note that the wettability of the sputter targets remained unchanged after breaking the Ar seal when compared with 600 h of exposure (Table 1).

**Table 1:** Apparent advancing (*θ*_a_) and receding (*θ*_r_) contact angles on the test surfaces upon breakage of Ar sealing and after 600 h of exposure to indoor laboratory air. The apparent contact angle for Er_2_O_3_ was not measurable (N.M.) because of the porosity of the sputtering target

Surface	Upon Ar seal breakage (t = 0)		600 h Air exposure		After solvent cleaning		After plasma cleaning	
	*θ*_a_	*θ*_r_	*θ*_a_	*θ*_r_	*θ*_a_	*θ*_r_	*θ*_a_	*θ*_r_
**CeO**_**2**_	112° ± 7°	7° ± 2°	111° ± 3°	8° ± 1°	85° ± 6°	7° ± 1°	15° ± 7°	<5°
**Er**_**2**_**O**_**3**_	N.M.	N.M.	N.M.	N.M.	N.M.	N.M.	N.M.	N.M.
**Yb**_**2**_**O**_**3**_	93° ± 2°	12° ± 9°	80° ± 1°	8° ± 2°	52° ± 13°	7° ± 1°	13° ± 5°	<5°
**Nb**_**2**_**O**_**5**_	63° ± 1°	8° ± 2°	56° ± 2°	<5°	48° ± 3°	6° ± 1°	26° ± 3°	<5°
**Cu**	120° ± 1°	34° ± 12°	127° ± 3°	10° ± 3°	71° ± 7°	7° ± 3°	25° ± 7°	<5°
**SiO**_**2**_	32° ± 2°	7° ± 1°	34° ± 3°	<5°	28° ± 2°	16° ± 1°	8° ± 2°	<5°

**Figure 1:**
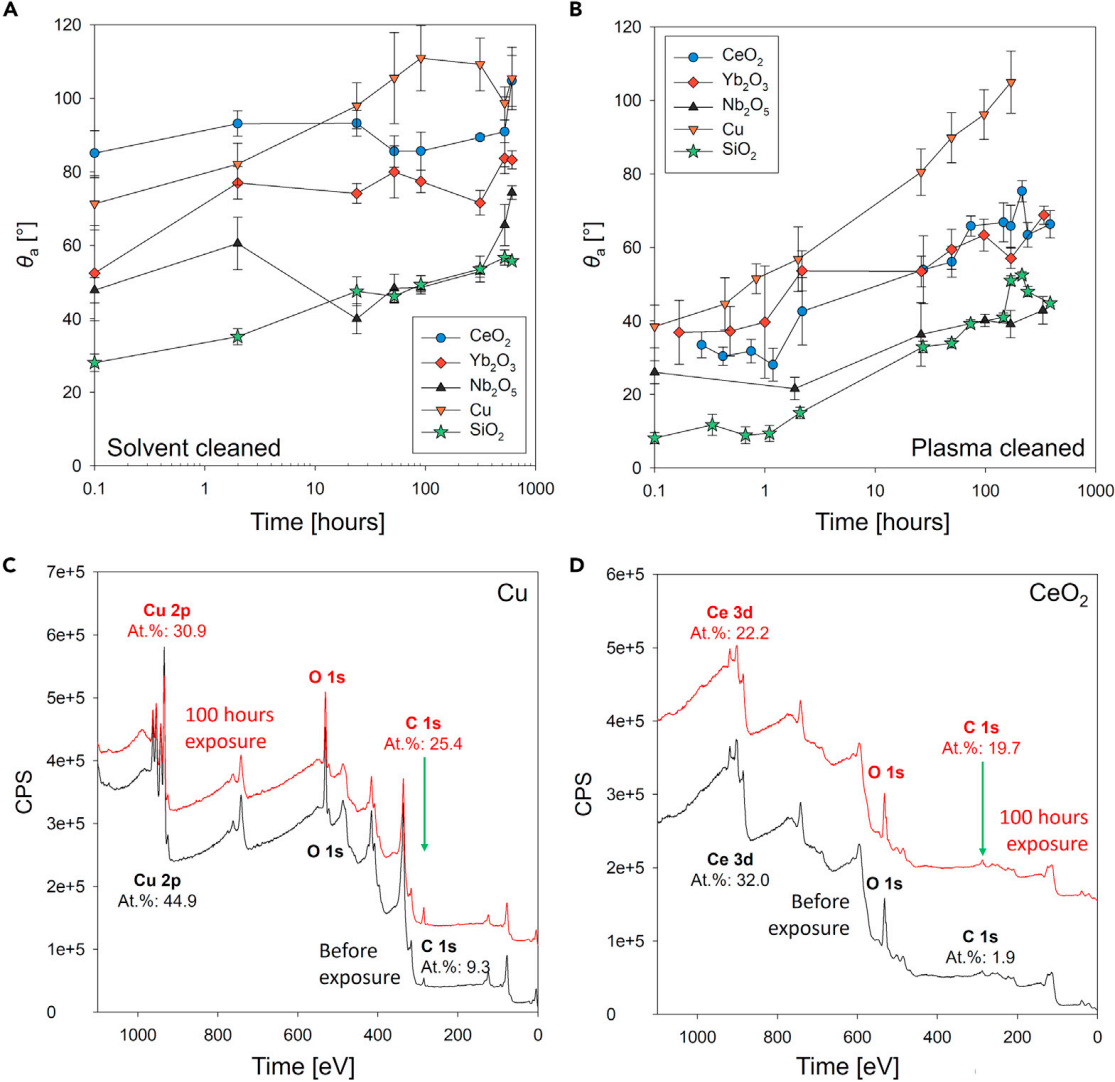
Transient water contact angle on REO sputter targets after solvent and plasma cleaning and exposure to air Apparent advancing contact angle (*θ*_a_) as a function of time exposed to air in Urbana, IL, USA, after (A) solvent cleaning and (B) plasma cleaning. Solid lines connecting different data points are trend lines meant to guide the eye. Error bars represent the standard deviation of *θ*_a_ from 10 independent measurements on different spatial locations on the same sample. X-ray photoelectron spectroscopy (XPS) spectra on the (C) Cu and (D) CeO_2_ surfaces after plasma cleaning (before exposure, black lines) and at 100 h of exposure (red lines) to ambient laboratory conditions.

After conducting contact angle measurements of the as-received samples, the surfaces were cleaned in a sonicating bath for 10 min with solvents in the flowing order: acetone, isopropyl alcohol (IPA), and deionized (DI) water, to remove adsorbed contaminants. On the solvent-cleaned surfaces, the apparent advancing contact angles of water droplets decreased but did not become highly wetting as sonication in organic solvents only removed physically adsorbed hydrocarbons, leaving the oxide layer and chemically adsorbed VOCs. Figure 1A shows the apparent advancing contact angle (*θ*_a_) as a function of time on the sputter targets after solvent cleaning. During the first 24 h of exposure to indoor laboratory air conditions, the advancing contact angle increased marginally. After 100 h of exposure, the advancing contact angle remained similar. After solvent cleaning and following exposure to indoor air for 600 h, the apparent advancing contact angle increased to *θ*_a,Cu_ = 116 ± 22°, *θ*_a,CeO2_ = 105 ± 7°, and = 83 ± 3° for Cu, CeO_2_, and Yb_2_O_3_, respectively, as shown in Figure 1A. The results demonstrate that cleaning with organic solvents could not completely remove hydrocarbons and VOCs already chemically adsorbed on the surfaces.

To better understand the effects of adsorbed materials on the surfaces and their impact on surface energy, we cleaned the samples with air plasma treatment at 45 W for 5 min. In general, plasma or UV exposure can remove physisorbed or chemisorbed organic contaminants from the surface (Taborelli, 2007). We used air as a process gas for plasma cleaning as oxygen plasma cleaning may significantly affect the surface oxidation state. Previous studies have reported that surfaces became superhydrophilic after Ar plasma cleaning by removing the hydrocarbon adsorbed on the surfaces (Lundy et al., 2017; Preston et al., 2014; Xu et al., 2021). Higher-power air plasma cleaning may provoke the surface to be terminated with -OH groups. However, narrow-band XPS spectra for the rare earth elements and oxygen remained identical showing negligible change before (new surface) and after the plasma cleaning. This indicates that -OH group generation was not the main reason for wetting increase in our results. As a consequence of air plasma cleaning, we were able to remove absorbed species from the surfaces and observed that all surfaces became highly hydrophilic as shown in Table 1. The surfaces were then left exposed to the indoor laboratory atmosphere for up to 600 h, and contact angles were measured at the same interval using the aforementioned procedure. Figure 1B shows the apparent advancing contact angle measured after plasma cleaning as a function of time.

To determine the mechanism of wettability transition, we conducted XPS analyses on the surfaces at different exposure times to provide further insights about the physicochemical properties at the free interface. Figures 1C and 1D show broadband XPS spectra for Cu and CeO_2_ after plasma cleaning and after 100 h of exposure to laboratory ambient conditions, respectively. For plasma-cleaned surfaces, the atomic percentage of carbon (C) on the Cu surface was 9% immediately after cleaning, increasing to 25% after 100 h of exposure. Copper is known to promptly oxidize once it is exposed to air and adsorbs VOCs resulting in increased contact angle (Yan et al., 2019; Zhou and Reed, 2018). Similarly, on the CeO_2_ surface, the atomic percent of C was 2% and 20% right after cleaning and after 100 h of exposure to laboratory conditions, respectively. Although the time between cleaning and XPS measurements was minimized, the presence of a C peak was inevitable, suggesting that the VOC adsorption on the surfaces is very rapid and spontaneous. Materials such as REOs are known to reduce under ambient conditions (Mullins, 2015); therefore, the REE-to-oxygen ratio did not match the theoretical ratio. However, we observed that the Ce 3d spectral region (875–925 eV) of CeO_2_ before and after exposure did not significantly change, indicating that the oxidation state of the surface remained the same as Ce(IV) (Paparazzo, 2018).

The increase in the advancing contact angle and the XPS C 1s peak after exposure allowed us to correlate the wettability transition and VOC adsorption. This was in contrast to the surface polarity hypothesis reported in the past work (Azimi et al., 2013; Khan et al., 2015). However, stronger evidence is required to rule out other mechanisms and to test our hypothesis that the wettability transition is solely governed by VOC adsorption. For example, as noted for Er_2_O_3_ surfaces, the contact angle was not measurable owing to porosity. Further AFM analysis of the sputtering target surface roughness revealed a height scan range of ±5 μm. Roughness affects wettability through the apparent contact angle measurement, which is governed by both surface energy and roughness.

To eliminate the effect of roughness and porosity on measured apparent contact angle, we sputter deposited REO thin films using a slow deposition rate (<0.05 Å/min) to grow 10-nm-thick films on polished silicon wafers. The base substrate material did not play a role in the wettability of the surface as a 10-nm thickness was enough to screen the substrate interaction potential (wetting transparency or translucency), which does not extend more than a few nanometers beyond the substrate interface (MacDowell, 2019; Stifter et al., 2000). To verify this, we conducted control experiments with a limited range of REOs by using sapphire as the substrate of choice (as opposed to a polished Si wafer). The sapphire measurements showed negligible change, indicating that the substrate beneath the deposited 10-nm-thick film plays no role on the measurements from an interaction potential perspective and only plays a role from a roughness perspective.

**Figure 2:**
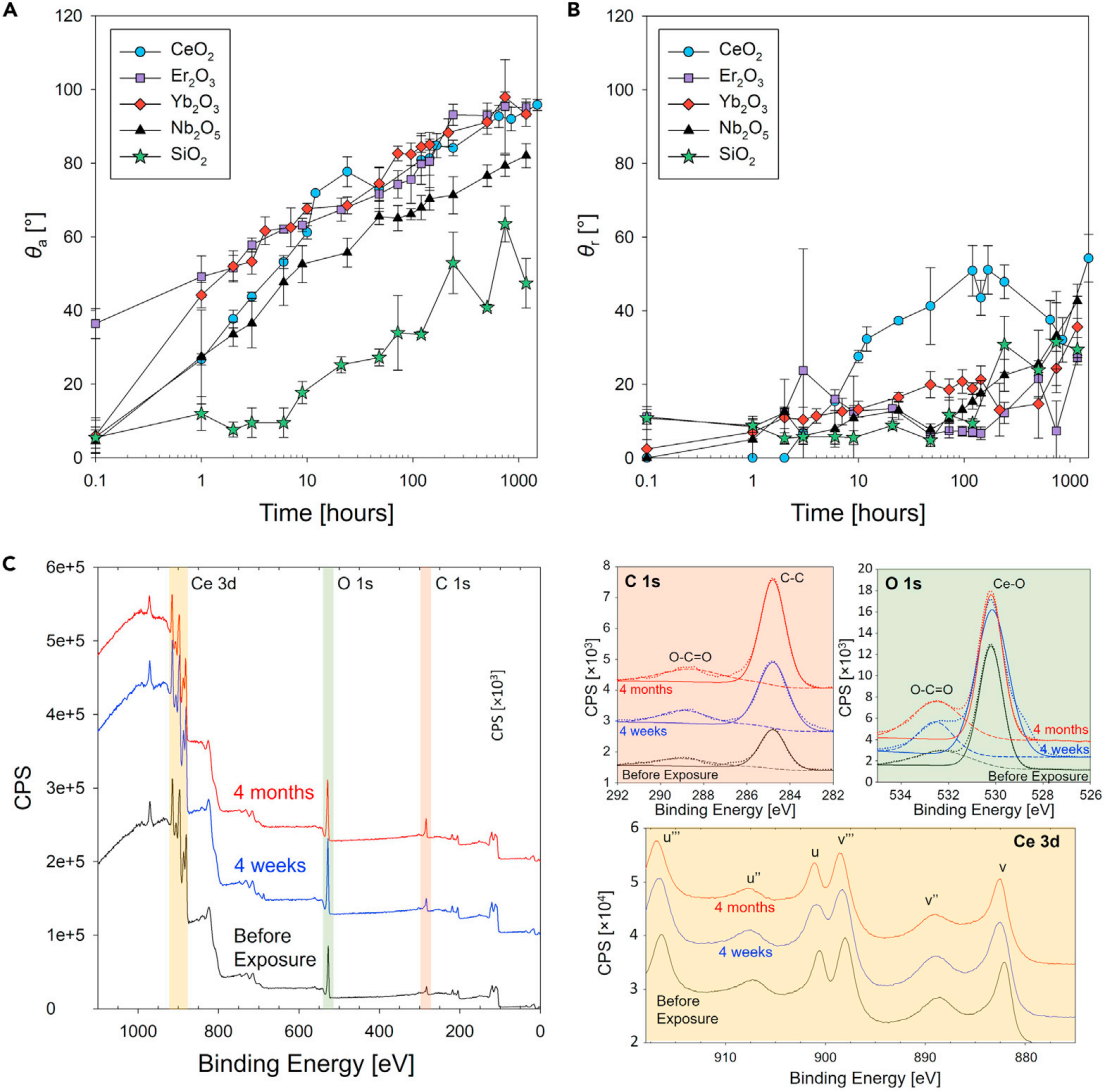
Transient water droplet contact angle on REO-coated Si wafers after long-term exposure to air Apparent (A) advancing (*θ*_a_) and (B) receding (*θ*_r_) contact angles as a function of time on CeO_2_, Er_2_O_3_, Yb_2_O_3_, Nb_2_O_5_ thin films deposited on polished Si wafers, as well as polished Si wafer controls. (C) Broadband and high-resolution narrow-band XPS spectra for C 1s, O 1s, and Ce 3d showing that the oxidation state of Ce did not change while the C content increased.

Further AFM measurements showed 1.8 ± 0.5-nm peak-to-peak roughness. Owing to the low roughness, the contact angle measured on the thin films represent the true intrinsic contact angle, which is solely affected by the surface energy of the material. Figures 2A and 2B show the apparent advancing and receding contact angles measurements on the sputtered test surfaces exposed to ambient laboratory conditions at different time intervals. The contact angle immediately after (within 5 min) film deposition revealed that the surfaces have a high surface energy, with *θ*_a_ < 10° in the case of CeO_2_ (*θ*_a,CeO2_ = 6 ± 5°) and Yb_2_O_3_ (*θ*_a,Yb2O3_ = 6 ± 5°). The advancing contact angles of REO surfaces (CeO_2_, Er_2_O_3_, and Yb_2_O_3_) increased with a similar trend over time as observed for the sputter target experiment (Figures 1A and 1B). The contact angles reached up to approximately 95° after 250 h of exposure and maintained the value until 1,000 h of exposure (*θ*_a,CeO2_ = 96 ± 1.5°, *θ*_a,Er2O3_ = 95 ± 2°, and *θ*_a,Yb2O3_ = 93 ± 3° at 1,000 h). Although the receding contact angle also increased with time, the rate of increase was slower than that of the advancing contact angle. As the advancing contact angle reached a plateau, the receding contact angle continuously increased with continued exposure. For Nb_2_O_5_ and the polished Si wafer, although both advancing and receding angles increased, the contact angle after 1,000-h exposure was lower compared with that of REO surfaces.

To provide further insights on the change in wettability of the surfaces based on their physicochemical properties, we investigated the broadband and narrowband XPS spectra for the elements of interest. Figure 2C shows the XPS broadband spectrum (left) with high-resolution narrow-band spectra for C 1s, O 1s, and Ce 3d immediately after plasma cleaning, after 4 weeks and 4 months of exposure. Over time, the XPS results clearly demonstrated the increase in hydrocarbon coverage on the surface. From the C1s spectra (282–292 eV), we observed a higher peak at 285 eV that corresponds to C-C bonding as the duration of exposure increased.

Table 2 summarizes the atomic percentage of elements retrieved from the entire spectra showing increasing carbon content from 21% to 38% over the 4-month exposure, whereas the Ce 3d:O 1s ratio was in a reasonable range of approximately 0.5. Furthermore, we observed an increasing trend of C-O bonding composition in the O1s spectrum. The ratio of C-O bonding with respect to Ce-O bonding increased from 0.26 to 0.55, indicating the presence of more oxygen atoms per carbon atom. The increased ratio of C-O bonding originates from either adsorbed VOCs or oxygen dissociated from Ce-O bonding further bonded to carbon. However, considering that the Ce 3d spectrum did not change, this indicates that the majority of oxygen atoms from the CeO_2_ surface remained bonded to Ce and the increase of oxygen stems from an external source such as VOCs. The increase in the C1s peak in the broadband spectrum and the increased C-C bonding in the narrow band spectrum (Table 2) demonstrates the presence of VOCs with increasing surface coverage of hydrocarbons as time increases. We observed a similar increase in carbon peaks on other REO surfaces (Er_2_O_3_ and Yb_2_O_3_) compared with the newly prepared surfaces (See Figure S1). We also found that the change in bonding composition changed more rapidly at earlier time periods during exposure, which corresponds to similar trends observed for wettability transition.

**Table 2:** Atomic percentage of Ce, C, and O and detailed spectral analyses of Ce 3d, C 1s, and O 1s from XPS on CeO_2_ surfaces before and after exposure to ambient laboratory air

Condition	Before exposure			4 weeks exposure			4 months exposure		
Elements	Ce 3d	C 1s	O 1s	Ce 3d	C 1s	O 1s	Ce 3d	C 1s	O 1s
**At. %**	27.56	21.02	51.41	20.54	26.94	52.52	20.17	38.21	41.62
**Detailed** **Peak** **Analyses**	C 1s	C-C	C-O	C 1s	C-C	C-O	C 1s	C-C	C-O
		70.99	29.01		76.55	23.45		77.52	22.48
	O 1s	Ce-O	C-O	O 1s	Ce-O	C-O	O 1s	Ce-O	C-O
		79.1	20.9		71.77	28.23		64.49	35.51

Although XPS and contact angle analyses demonstrate that the transient surface chemistry changes are likely due to VOC adsorption, the results do not provide a complete picture of the adsorption mechanisms and kinetics. To gain a better understanding on the adsorption process, and to provide a second test of our hypothesis, we conducted experiments in highly controlled conditions with a light hydrocarbon atmosphere containing alkanes with six to ten carbon atoms from hexane (C_6_H_14_) to decane (C_10_H_22_) to simulate airborne VOCs. The aliphatic hydrocarbons have similar molecular weights with the major species of VOCs in indoor and outdoor environments (Gligorovski and Abbatt, 2018; Valsaraj, 1994). We placed CeO_2_ films deposited on the polished Si wafers in a large sealable glass laboratory bottle along with the hydrocarbon in a separate open vial placed next to the samples. Samples were inserted immediately after fabrication. The glass bottle was kept at room temperature, after which we repeated the same experimental procedure for contact angle measurements and surface analyses at different intervals of time.

**Figure 3:**
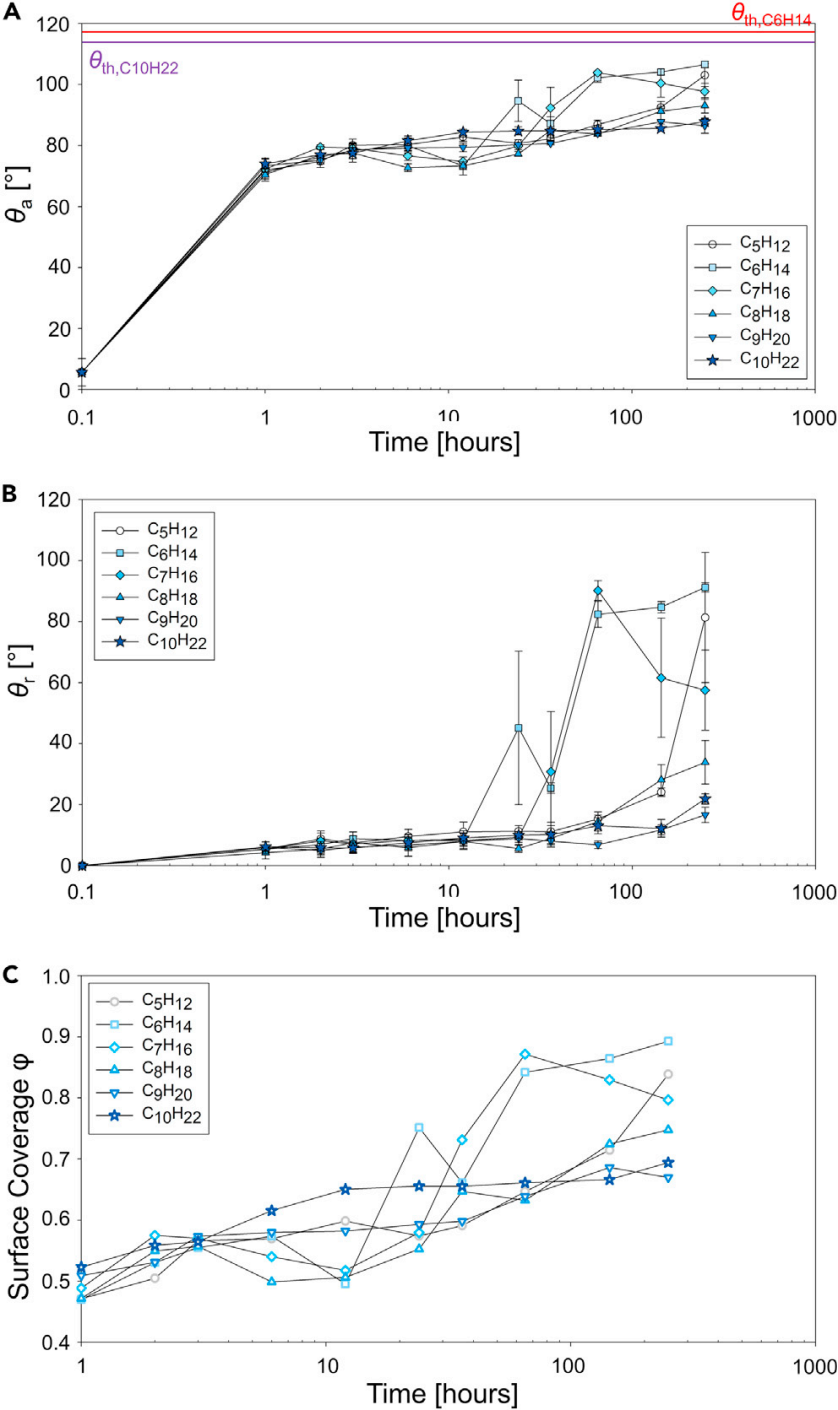
Transient contact angle on REO-coated Si wafers after exposure to a hydrocarbon saturated atmosphere Apparent (A) advancing and (B) receding water droplet contact angles as a function of time on sputtered CeO_2_ surfaces left exposed in a saturated hydrocarbon (C_6_H_14_ to C_10_H_22_) atmosphere. The horizontal solid lines in (A) indicate the calculated maximum contact angle on a flat surface with homogeneous alkane chemistries for C_6_H_14_ (red line) and C_10_H_22_ (purple line). (C) Surface coverage (*φ*) was calculated using the maximum contact angle.

Figures 3A and 3B show the apparent advancing and receding contact angles on sputtered CeO_2_ surfaces exposed to the alkanes as a function of time. The initial increase in the advancing contact angle on the surfaces was immediate and was independent of the length of the hydrocarbon studied. The advancing contact angles increased rapidly within an hour of exposure and continued to increase gradually during the subsequent 11 h of exposure. However, after 12 h of exposure, the contact angle on the surfaces exposed to C_9_H_20_ and C_10_H_22_ remained almost constant, with continued increase on surfaces exposed to C_6_H_14_, C_7_H_16_, and C_8_H_18_. We observed a similar trend in receding contact angles for similar time frames, with receding contact angles on the surfaces exposed to lighter hydrocarbons sharply increasing when compared with other samples.

To study the surface coverage of hydrocarbons (Lundy et al., 2017), we obtained estimates based on surface chemistry and wettability measurements. We first calculated the maximum theoretical contact angle using the Young-Dupre equation (Equation 1) where *θ* is the experimentally measured apparent advancing contact angle, cos *θ*_th_ is theoretical contact angle calculated from the interfacial energies, and *γ*_s1_, *γ*_sv_, and *γ*_1V_ are the interfacial energies of the surface-liquid, surface-vapor, and liquid-vapor interfaces:

(Equation 1) $$\cos\;\theta =\frac{\gamma_{\text{sl}}\;-\;\gamma_{\text{sv}}}{\gamma_{\text{lv}}}\;,$$

(Equation 2) $$\varphi_{t\;=\;t1}=\frac{\left(\frac{\cos\;\theta_{t=t1}^{app}\;}{\cos\;\theta_{a,t=0}^{app}\;}\right)}{\left(\frac{\cos\;\theta_{\text{th}}\;}{\cos\;\theta_{a,t=0}^{app}\;}\right)}\;.$$

Here, we used the apparent advancing contact angle in lieu of the equilibrium contact angle to better characterize the wettability and its transition. As discussed in the methods section, the equilibrium contact angle is an ambiguous quantity especially when the surface has a high contact angle hysteresis, which is the case here during the early stages of the wettability transition. The apparent advancing contact angle increases as hydrocarbon adsorption ensues, denoting the wettability transition better. Studies using solid-vapor interfacial energy measurement also adopt the same approach using the apparent advancing contact angle for the wettability characterization (Ma et al., 2020a, 2021; Zhao et al., 2021).

Our analysis assumes full surface coverage with hydrocarbons. Hence, we adopted hydrocarbon properties for the surface phase in Equation (1). From the measured advancing contact angle, we calculated the surface coverage (*φ*) using Equation (2) to examine how conformally the surfaces adsorbed hydrocarbons. The interfacial energies used in our analysis are summarized in Table 3.

**Table 3:** Interfacial tensions of n-alkanes with air and water. The theoretical water contact angle on alkane-adsorbed surfaces was calculated using Equation (1)

Parameter	C_6_H_14_	C_7_H_16_	C_8_H_18_	C_9_H_20_	C_10_H_22_
γ_CxHy–air_ [mN/m]	18.3	20.05	21.55	22.7	23.7
γ_CxHy–water_ [mN/m]	51.4	51.9	52.5	52.4	53.2
*θ*_th–CxHy_ [°]	117.0	115.9	115.2	114.1	113.9
P_sat_ at 25°C [kPa]	17	4.6	1.33	0.59	0.17

Figure 3C plots the hydrocarbon surface coverage (*φ*) on sputtered CeO_2_ surfaces calculated using Equation (2) with the theoretical advancing contact angle. The model suggests a rapid increase for heavier hydrocarbons (C_8_H_18_, C_9_H_20_, and C_10_H_22_). The surface coverage gradually increases in time following a similar qualitative trend as observed for the apparent advancing contact angle. More importantly, surface coverage analysis allows us to compare the kinetics and the extent of adsorption as a function of the different alkane atmospheres.

We initially hypothesized that surfaces exposed to lighter hydrocarbons would display higher surface coverage owing to their higher vapor pressure (Table 3). Interestingly, at early stages, the coverage on the surfaces exposed to heavier hydrocarbons such as nonane (C_9_H_20_) or decane (C_10_H_22_) was higher than the ones exposed to lighter hydrocarbons. The sticking coefficients for both physisorption and chemisorption are higher for heavier hydrocarbons (Cushing et al., 2010; Wetterer et al., 1998). However, this trend lasted only during the initial 12 h, after which the contact angle of surfaces with lighter hydrocarbons rapidly increased.

Unlike the sharp increases in advancing contact angle, we observed strong pinning at the contact line in early exposure times resulting in no significant increase in the receding contact angles as shown in Figure 3B. The receding contact angle started to increase between 12 and 100 h of exposure when the advancing contact angle and surface coverage suddenly increased. The increases in the receding contact angle for the surfaces exposed to C_6_H_14_ and C_7_H_16_ were more rapid when compared with the gradual increases observed on the surfaces that had adsorbed heavier hydrocarbons. This trend corresponded well with the sudden increase in advancing contact angle, which may reflect the change in adsorption dynamics naturally occurring as no change was made in the experimental environment. Previous studies on physisorption on Au or Pt surfaces have shown that sticking coefficients for alkanes lighter than octane (C_8_H_18_) approach zero at room temperature (Wetterer et al., 1998). Physisorption of alkanes are mainly governed by van der Waals forces, of which heavier molecules have larger interactions when compared with lighter molecules. Similarly, the sticking coefficient of dissociative adsorption (chemisorption) is an order of magnitude lower when an alkane has one less carbon in its chain (Cushing et al., 2010). Owing to limited physisorption for lighter hydrocarbons at room temperature, the contact angle increase on the surfaces exposed to lighter hydrocarbons should initially rely on chemisorption. Our results point to chemisorption as the dominant surface adsorption mechanism at early stages.

**Figure 4:**
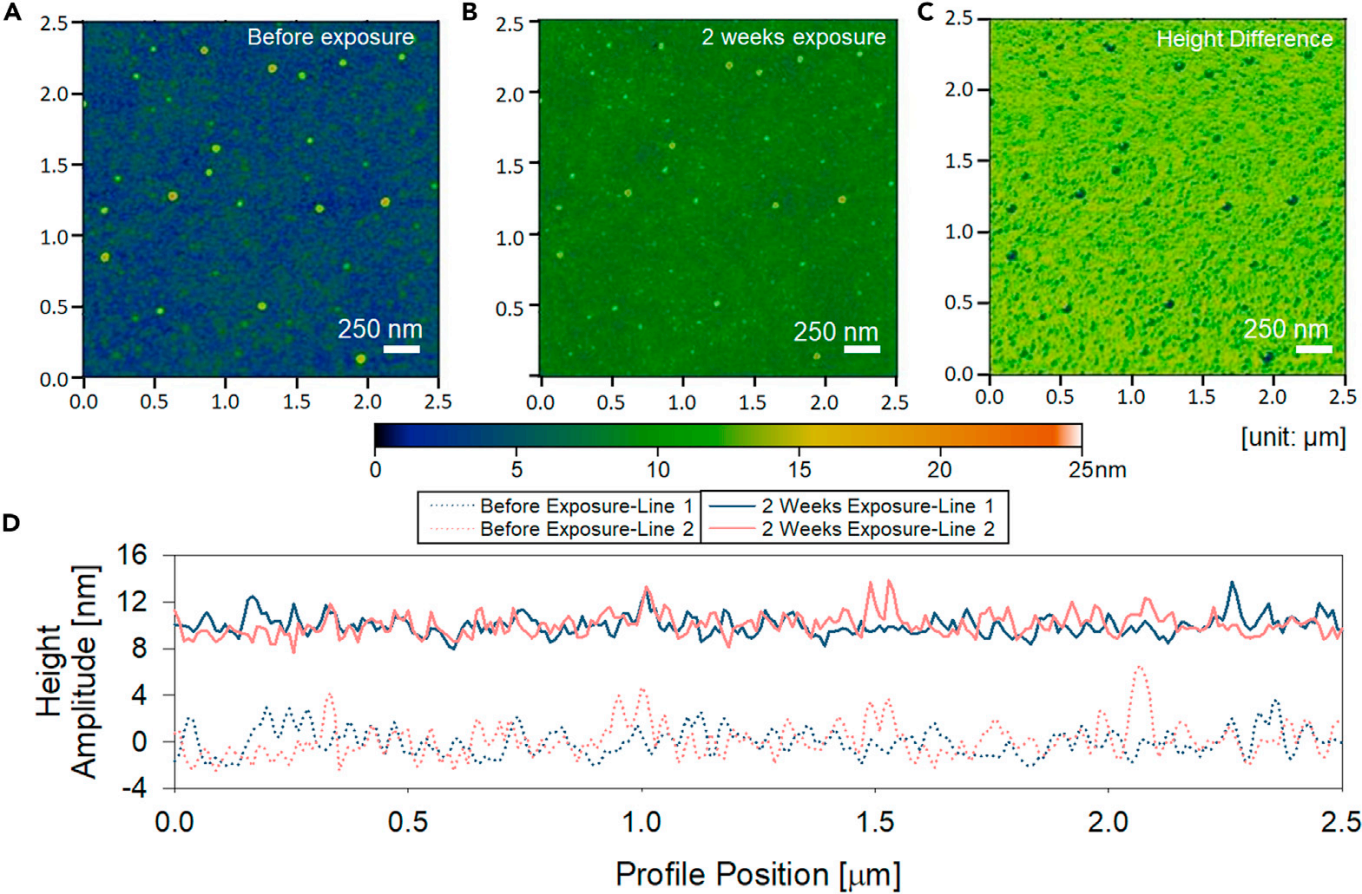
Change in surface topographical profile on a CeO_2_-coated Si wafer due to hydrocarbon adsorption Atomic force microscopy (AFM) images of a CeO_2_ film deposited on a polished Si wafer (A) before exposure, (B) after 2 weeks of exposure in a hexane (C_6_H_14_)-saturated atmosphere, and (C) the height difference from scan results in (A) and (B). (D) AFM height amplitude profiles on a line scan. The dotted and solid lines are profiles on surfaces before and after exposure, respectively. Two separate lines were scanned for each time point, delineated by Spot 1 or Spot 2. The scan area for all scans was 2.5 μm × 2.5 μm.

To observe adsorption and surface coverage dynamics directly, we used AFM on identical spatial locations at different exposure times (Külah et al., 2017). We fabricated a test Si wafer surface with number markers etched to identify spatial location to enable AFM of the same areas. Figures 4A and 4B show the height amplitude profile of the sputtered CeO_2_ surface before exposure and after two weeks of exposure to hexane (C_6_H_14_) in the controlled atmosphere. Figure 4C represents the height difference, which depicts the change in height profile due to hydrocarbon adsorption. We observed that there was a change in the height of the CeO_2_ surface of approximately 10 nm, which is similar to observations in past studies (Külah et al., 2017). The height amplitude on the entire surface increased, whereas the peak-to-peak differences in the height amplitude were negligible where dust particles reside (dark dots in Figure 4C). Figure 4D displays the surface profiles of two different line scans on the surface before and after exposure, which are extracted from Figures 4A and 4B. The ridges and valleys on the AFM profiles before and after exposure matched well with a minor lateral shift. The agreement between the profiles and the increased height on the entire surface show hexane was adsorbed conformally. The root-mean-square (RMS) roughness for the scanned area was 1.60 nm for a sample before exposure and 0.72 nm for 2 weeks exposure to C_6_H_14_ while the entire background height increased owing to adsorption.

### Adsorption mechanism: chemisorption versus physisorption

Our experiments demonstrate the ability to correlate wettability transition to VOC adsorption. Although XPS identifies the presence of foreign elements and chemical bonding, it has difficulty obtaining the molecular structure of VOCs present on the surface. To gain a better understanding of the molecular structure of VOC species on the surface, we conducted ToF-SIMS on CeO_2_ thin film surfaces deposited on the polished Si wafers.

**Figure 5:**
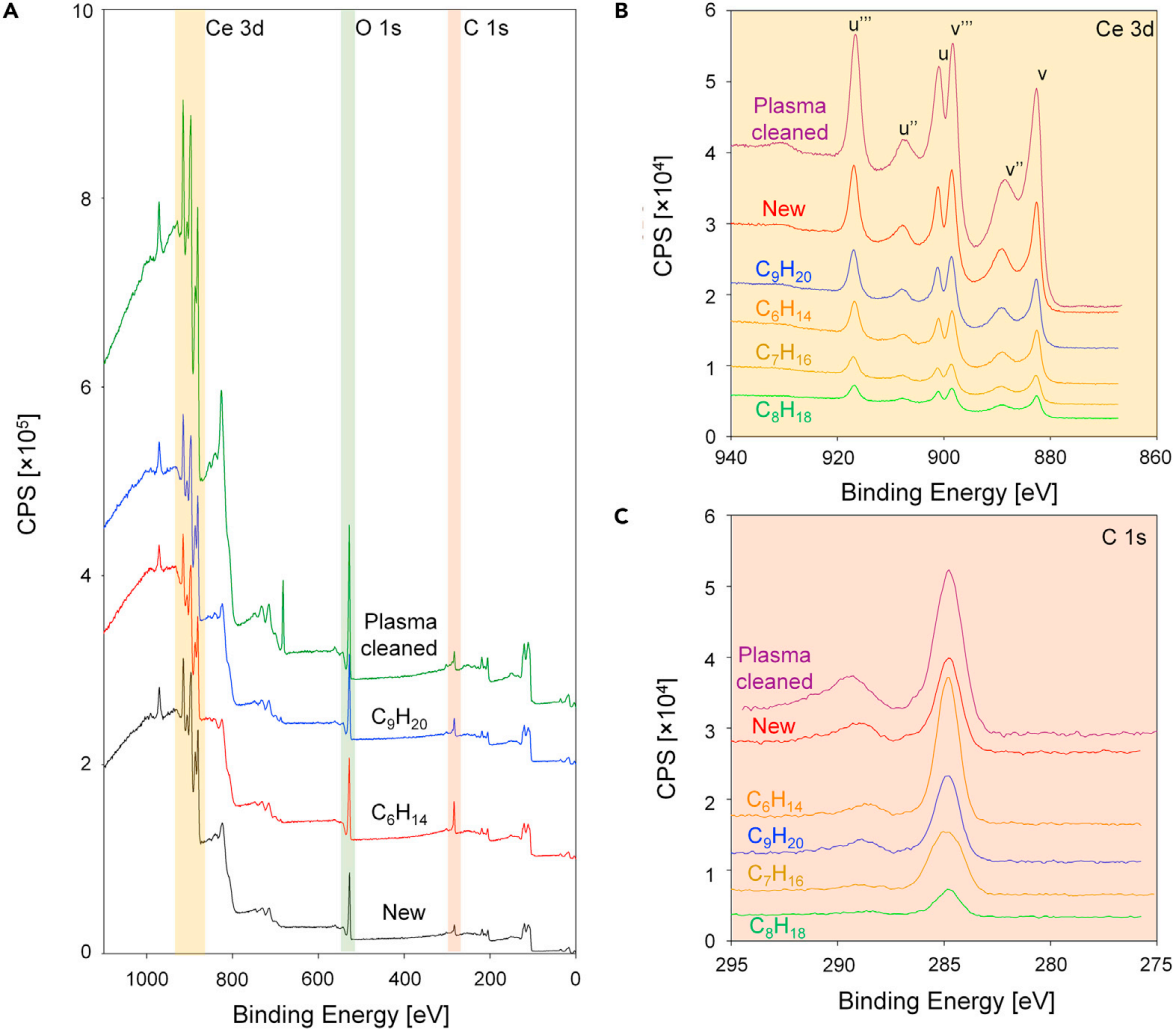
Surface chemistry analyses using XPS and ToF-SIMS on CeO_2_ exposed to a hydrocarbon saturated atmosphere (A) Broadband XPS spectra on a freshly fabricated CeO_2_ film, CeO_2_ surface after plasma cleaning, and a plasma-cleaned CeO_2_ surface after C_6_H_14_ and C_9_H_20_ exposure for 1,000 h. (B and C) XPS high-resolution spectra obtained for (B) Ce 3d and (C) C 1s to verify the oxidation state and hydrocarbon adsorption after exposure to aliphatic hydrocarbons, respectively.

Figure 5 and Table 4 show the surface chemistry of CeO_2_ obtained from XPS and ToF-SIMS. Figures 5A–5C show the broadband and narrow-band XPS spectra of freshly deposited, plasma-cleaned, and hydrocarbon-adsorbed CeO_2_ surfaces in the controlled alkane atmosphere. Adsorption of VOCs on freshly fabricated and plasma-cleaned CeO_2_ surfaces was inevitable because of the rapid adsorption kinetics, as evidenced by the presence of C on the surface (Figure 5A at 285 eV). However, according to the Ce 3d spectra in Figure 5B and the composition data provided in Table 4, we confirmed that the oxidation state of ceria was Ce(IV) during exposure or after plasma cleaning. While maintaining its oxidation state, the ceria surface adsorbed hydrocarbons from the saturated alkane atmosphere that resulted in increased C content from ≤10% to >20%. Interestingly, the ratio of C 1s spectra for C-C (284.8 eV) increased compared with O=C-O (288.6 eV) on most surfaces. On the alkane-adsorbed surfaces, C-C (284.8 eV) bonding contributed to ≈90% of the C content on the surface due to the majority of adsorbed molecules being composed of C and H atoms.

**Table 4:** Broadband and high-resolution XPS results on a fresh CeO_2_ film, plasma-cleaned CeO_2_ films exposed to alkane environments with different carbon chain lengths for 2 months, and plasma-cleaned surfaces after hydrocarbon exposure. High-resolution spectra were obtained in the range of 280–295, 525–540, and 870–925 eV for C1s, O1s, and Ce3d, respectively. All presented values are percentages (%)

Spectra [eV]		New Surface	CeO_2_-C_6_H_14_	CeO_2_-C_7_H_16_	CeO_2_-C_8_H_18_	CeO_2_-C_9_H_20_	CeO_2_-C_10_H_22_	Plasma cleaned
**Ce 3d** **(875–918 eV)**		32.5	29.9	28	29.1	26.4	29.5	31.8
**C 1s** **(282–290 eV)**		6.8	24.3	27.2	26.9	29.4	25.3	10.5
**O 1s** **(527–535 eV)**		60.7	45.8	44	44	44.2	45.2	57.7

**Narrow band spectra for each element**								
**C 1s**	**O-C=O** **(288.6)**	18.2	9.4	7.3	8.9	9.5	14.9	23.3
	**C-C** **(284.8)**	81.8	90.6	92.7	91.1	90.5	85.1	76.7
**O 1s**	**O-C=O** **(533.8)**	24.2	25.9	21	22.8	24.8	23.6	19.7
	**O-Ce** **(531.2)**	75.8	74.1	79	77.2	75.3	76.4	80.3
**Ce 3d**	**u’’’** **(916.7)**	10.4	10.1	9.9	10.6	10.1	10.2	10.1
	**u’’** **(907.4)**	23.1	26.9	24.9	24.7	26.9	25	26.2
	**u** **(901.0)**	8.9	8	8.9	8.4	8	8.4	8.7
	**ν‴** **(898.4)**	15.1	13.7	14	14.6	13.7	14.2	12.2
	**ν’’** **(888.8)**	28.5	28.3	28.5	28.7	28.3	28.9	27
	**ν** **(882.6)**	14	13.1	13.8	13.1	13.1	13.3	15.8

We expected that surfaces exposed to heavier hydrocarbons would have higher percentages of C-C bonding when compared with ones exposed to lighter hydrocarbon because of the longer aliphatic chains. However, the high-resolution XPS spectra showed the contrary, with heavier hydrocarbon-exposed samples having lower percentage of C-C bonding when the atomic concentration of carbon is relatively similar. From these results, we infer that dissociative adsorption occurs on the surface and dominates VOC adsorption behavior over physisorption as heavier hydrocarbons have a smaller activation energy of dissociation when compared with lighter hydrocarbons (Lavrich et al., 1998).

**Figure 6:**
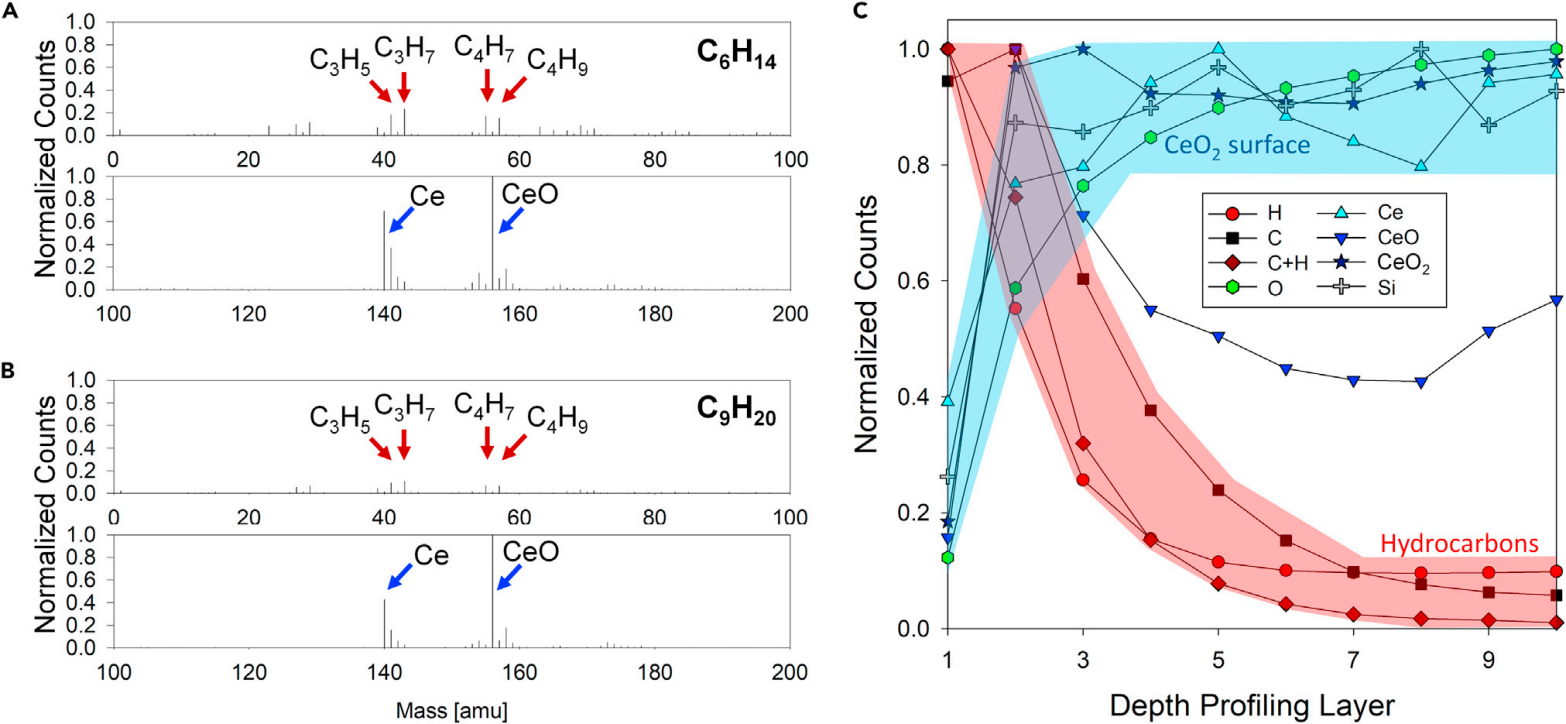
ToF-SIMS results for secondary ion species and depth profile on CeO_2_ ToF-SIMS results of (A) hexane (C_6_H_14_)- and (B) nonane (C_9_H_20_)-exposed CeO_2_ surfaces for positive ions normalized by CeO (156 amu) ion count. (C) Depth profile of secondary ions detected as a function of the number of layers removed from the surface.

The ToF-SIMS analyses support the VOC adsorption hypothesis and enabled quantification of the chemical species adsorbed on the surface. Figures 6A–6C and Table 4 demonstrate that surfaces exposed to lighter hydrocarbons (i.e., C_6_H_14_) have higher counts of C_x_H_y_-positive secondary ions compared with heavier hydrocarbons (i.e., C_9_H_20_). To understand the chemical species beneath the top exposed interfaces, we conducted depth profiling ToF-SIMS on a CeO_2_ surface exposed to hexane (C_6_H_14_, Figure 6C). The depth profile shows that the adsorbed hydrocarbon layer exists at the interface between air and CeO_2_ as the C, H, and C + H ion counts decreased significantly after the initial layers. The hydrocarbon-related ion counts became very low after the 10^th^ profiling layer. However, ions from the constituent of CeO surfaces such as Ce, O, CeO, and CeO_2_ increased as hydrocarbon layers were sputtered from the surface by the primary Cs^+^ ions. The depth profiling layer thickness was approximately 1 nm per layer corresponding well with the AFM scan results, which suggested a 10-nm-thick layer of adsorbed hydrocarbons after 2 weeks of exposure.

Although the contact angle measurements and surface analyses demonstrate the importance of hydrocarbon adsorption on surface energy and wettability, they fail to identify the fundamental mechanism of adsorption, i.e., physical adsorption by intermolecular attraction or chemical adsorption by chemical bonding. To better examine the adsorption phenomena, we conducted experiments to isolate physisorbed hydrocarbons from the surface as much as possible. We cleaned surfaces that had undergone hexane (C_6_H_14_) adsorption with different cleaning methods and doses of air plasma cleaning controlled by duration of exposure. After cleaning the surfaces, we repeated the same hydrocarbon exposures in the same controlled alkane environment and the advancing contact angles were measured to examine whether different adsorption behavior changed depending on the different hydrocarbon molecules already adsorbed on the surface.

**Figure 7:**
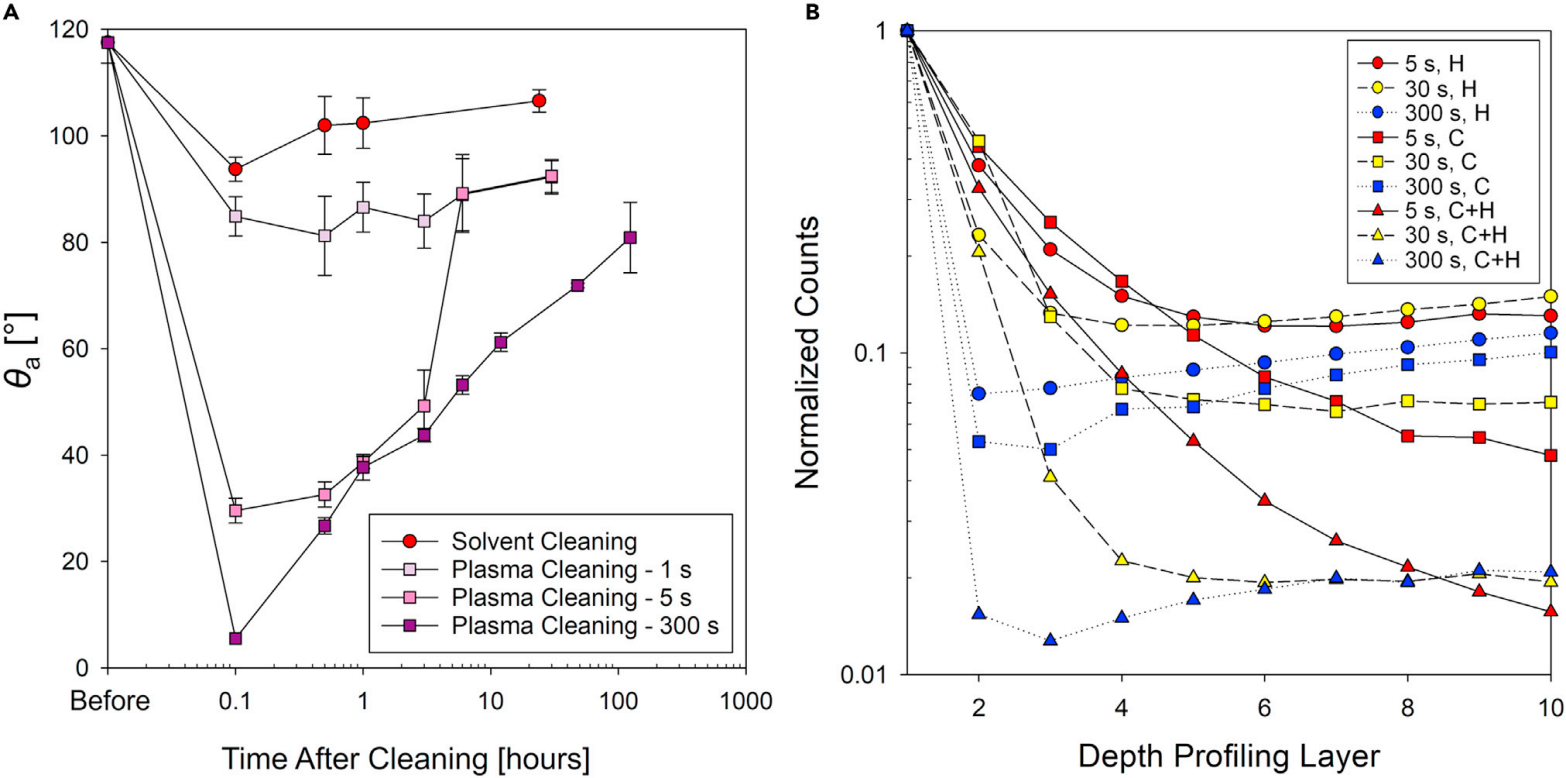
Contact angle recovery after surface cleaning and ToF-SIMS analysis for different plasma durations (A) Advancing contact angle measured on CeO_2_ films after solvent and plasma cleaning with different doses controlled by time duration. For detailed cleaning methods, refer to the experimental section. (B) Normalized secondary ion counts from ToF-SIMS for hydrogen (H), carbon (C), and C + H ions using depth profiling on surfaces cleaned with different plasma durations.

Figure 7A shows the advancing contact angle measured after 1, 5, and 300 s after plasma cleaning, and solvent cleaning. After 300 s of plasma treatment, the CeO_2_ surface became very hydrophilic, displaying the greatest decrease in the advancing contact angle from 118 ± 4° (at 0 h) to ≈5° (at 0.1 h in Figure 6A). In contrast, surfaces treated for shorter durations showed limited decreases in advancing contact angles to 30 ± 2° and 85 ± 4° for 5- and 1-s plasma exposures, respectively. The decrease in the contact angle with respect to different plasma cleaning doses suggests that hydrocarbons adsorbed on the surface are removed to a different extent depending on plasma exposure time. The cleaned surfaces were then further exposed to the same alkane environment again to examine the difference in hydrocarbon adsorption dynamics due to the difference in surface chemistry. Solvent cleaning with organic solvents relies on micelle formation or van der Waals forces to detach physically adsorbed contaminants (Taborelli, 2007). Therefore, solvent cleaning only removes physisorbed hydrocarbons or contaminants existing at the top exposed interface. Hence, solvent cleaning leaves a thicker organic contaminant layer compared with plasma cleaning (Mantel and Wightman, 1994). Furthermore, plasma cleaning activates hydroxide sites on metallic surfaces, resulting in a higher surface energy of the plasma-cleaned surface compared with a solvent-cleaned surface. The wettability measurements and surface analysis after solvent cleaning (Figure 7) prove the presence and effect of physisorbed molecules on the surface wettability, as well as further elucidates the role of chemisorbed hydrocarbons.

Interestingly, we observed fast contact angle recovery after cleaning (Figure 7A) compared with the initial increase on freshly prepared surfaces (Figure 2A, airborne VOCs, or Figure 3A, controlled C_6_H_14_). The solvent-cleaned surface recovered its contact angle within the shortest period (∼30 min) as the contact angle decrease by solvent cleaning was very limited compared with the plasma cleaning. Plasma cleaning removes chemisorbed hydrocarbons and leaves more active sites such as -OH radicals on the surfaces, which promotes adsorption of hydrocarbons on the surface. An instant plasma treatment (1 s) showed very similar trends with the solvent-cleaned surface, whereas surfaces cleaned with longer plasma treatments (i.e., 300 s) made the surfaces similar to the freshly prepared samples. Although the wettability recovery of surfaces cleaned with lower doses of plasma cleaning took longer than for solvent-cleaned surfaces, the rate of re-adsorption of hydrocarbons on the plasma-treated surfaces was remarkably fast, as shown by the rapidly increasing contact angle.

The results indicate that the mechanism governing the adsorption of hydrocarbon molecules is a delicate balance between both chemisorption and physisorption (De Moor et al., 2009; Lavrich et al., 1998; Lundy et al., 2017). Since the adsorption of gas molecules on solid surfaces is spontaneous, it is very sensitive and difficult to clearly distinguish how the molecules are adsorbed to the adsorbent. Previous studies have reported physisorption enthalpies of alkanes in the range of 40–60 kJ/mol, with a chemisorption enthalpy in the range of 120–150 kJ/mol.

The mechanism can also be understood with analogy to self-assembled monolayer (SAM) adsorption dynamics of fluorosilanes on oxide surfaces (Paxson et al., 2014). SAMs form a chemical bond with the substrate using their silanol group and the hydroxyl group on the surface during deposition. The silanol bonding can be broken during plasma cleaning (Wang et al., 2011), and the coated surface can lose its hydrophobicity. However, the silane coating is chemically durable to solvents such as acetone and IPA (Montemor, 2014). Similarly, the hydrocarbon-free pristine REOs as well as plasma-cleaned REOs are hydrophilic. After adsorption of VOCs, the REOs become hydrophobic because of the lower surface energy of hydrocarbons. Meanwhile, solvent cleaning only dissolves physisorbed hydrocarbon molecules, leaving the chemisorbed hydrocarbons on the surface. Subsequently, the surface covered with chemisorbed hydrocarbons can further promote physisorption of organic compounds much faster than the hydrocarbon-free surface as shown in the other studies regarding chemisorption-mediated physisorption (Xu et al., 2021).

Figure 6B supports the combined adsorption mechanism by demonstrating the differences in H and C + H cations detected between samples cleaned in the plasma chamber for different durations. The samples were solvent cleaned just prior to introduction to the high-vacuum environment of the ToF-SIMS chamber. The results show that significant amounts of hydrogen and hydrocarbon negative ions were detected from the several initial layers on the surface treated with plasma for shorter times such as 5 and 30 s compared with a thoroughly cleaned surface for 300 s. After the fifth layer, the levels of hydrogen and hydrocarbon ions were similar on all the surfaces as the ions sputter the top layer of the cerium oxide-adsorbed hydrocarbon from airborne atmosphere.

**Figure 8:**
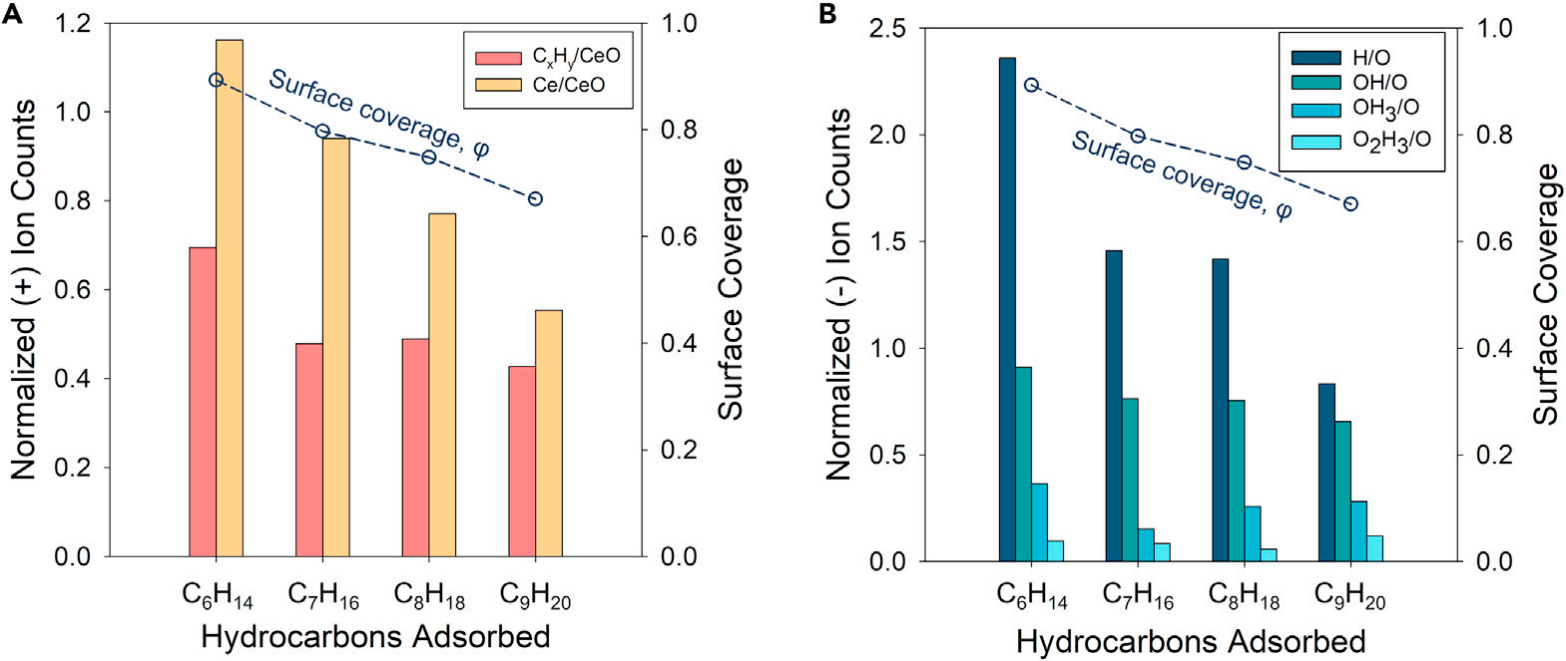
Surface coverage of hydrocarbons adsorbed on a CeO_2_ surface Normalized ToF-SIMS results of (A) positive ions detected normalized by CeO^+^ ion counts and (B) negative ions detected normalized by O ion counts. Histogram results indicate normalized ion counts (left axis), whereas the dotted black line indicates the surface coverage (right axis). C_x_H_y_ ions account for positive ions composed of hydrogens and less than five carbons (x < 5). The surface coverage of each hydrocarbon-exposed surface was calculated from Equation (2) and the contact angle measurement data after 1,000 h of exposure for each specific saturated alkane atmosphere.

Figure 8 shows further ToF-SIMS results that provide insight into the nature of chemical adsorption of organic compounds (for the detailed table, see Table S1). The positive ion counts were normalized with CeO^+^, whereas the negative ion counts were normalized with O^−^, as they are representative species of the original CeO surface. We detected a larger number of hydrocarbon-based (C_x_H_y_) positive ions on the surfaces exposed to lighter hydrocarbons. It is difficult to confirm and specify the amount of C_x_H_y_ positive ions associated to physisorbed hydrocarbons or chemisorbed ones, even though we attempted to minimize the amount of hydrocarbon adsorbed on the surface before introducing samples into the high-vacuum environmental chamber for ToF-SIMS analyses. However, Figure 8A shows more Ce ions were detected compared with CeO ions on the surface exposed with lighter hydrocarbon-saturated environments. The higher normalized ion counts suggest that CeO at the outermost surface is reduced while the oxygen formed chemical bonds with the adsorbed hydrocarbons. This corresponds to a larger C_x_H_y_ ion count obtained from the surface exposed to lighter hydrocarbons showing more hydrocarbons were chemically adsorbed on the REO surface.

We observed similar results supporting the combined adsorption hypothesis from the negative ion spectroscopy data of Figure 8B. Negative ion analysis showed a similar trend with the positive ion results showing that H and OH negative ions detected, normalized by O negative, were in a decreasing trend as the contact angle and surface coverage decreased. Interestingly, the C_x_H_y_ positive and OH negative ion results for heptane- and octane-exposed surfaces were similar, which indicates that chemisorption of hydrocarbons is likely prior to physisorption at later stages. Previous studies have suggested that VOCs mainly consist of lighter hydrocarbons, which can be both chemisorbed and physisorbed on metallic oxide surfaces (Yan et al., 2019, 2020). The results presented here confirm this hypothesis.

## Discussion

The extensive surface chemistry analyses and temporal contact angle measurements on REOs conducted here point to REO intrinsic hydrophilicity with VOC hydrocarbon adsorption governing wettability transition to hydrophobicity. Throughout our experiments, we explored the adsorption mechanisms, demonstrating that adsorption is based on both chemisorption and physisorption, with neither one having exclusivity to wetting transition. Differences in the wettability recovery after plasma and solvent cleaning demonstrated that chemisorption assists physisorption. The experiments conducted in the controlled alkane atmospheres elucidated key differences in adsorption and wettability transition dynamics with respect to the number of carbon atoms in the alkane.

Our findings offer a better understanding of the effects of adsorption of airborne VOCs in controlled laboratory conditions and under different VOC environments in addition to supporting the environmental ubiquity of these VOCs (Cha et al., 2017). The majority of indoor VOCs are aliphatic hydrocarbons such as alkanes or aromatic hydrocarbons such as benzene or toluene, which may originate from various sources such as paints, adhesives, solvents, and other chemicals. The largest contribution to indoor VOCs are aromatic hydrocarbons and shorter aliphatic chains with less than 10 carbon atoms (Rösch et al., 2014). VOC adsorption to REO surfaces is a more complex phenomenon than the controlled atmosphere experiments conducted here as many chemical species compete with each other via different adsorption mechanisms, kinetics, and concentrations. However, it is practically impossible to isolate each species and control conditions perfectly to simulate VOC adsorption. However, based on our results, heavier hydrocarbons chemisorb faster than lighter hydrocarbons initially on pristine samples, and subsequently lighter hydrocarbons or other abundant species will be further physisorbed on the surface. This adsorption behavior changes the surface wettability of REO surfaces simultaneously, resulting in significant changes in transport phenomena.

Many researchers have reported enhanced durable condensation on REO surfaces under high temperature and mechanically abrasion conditions. Furthermore, since REOs have a higher thermal conductivity (≈10 W/(m∙K)) when compared with polymer-based hydrophobic coatings (≈0.05 to 1 W/(m∙K)), REO-based hydrophobic surfaces have drawn interest as a candidate material to enhance condensation and boiling heat transfer. However, past studies have focused on developing the methods to fabricate REO coatings based on the hypothesis that REOs are intrinsically hydrophobic. However, as demonstrated in our results, we show that hydrophobicity stems from the adsorption of VOCs. Thus, more work is needed to better understand the mechanisms of VOC adsorption during condensation and other use conditions. It will be very important to fully investigate the detailed mechanism to ensure that the hydrophobicity of REOs is thermally and chemically robust and self-healing in practical condition by replenishing the adsorbed layer.

Adsorption-based non-wetting coatings can be extended to a host of other materials including metals, oxides, and 2D materials. Recently, many researchers have shown interest in VOC adsorption on these alternate surfaces with a focus on their ability to affect hydrophobicity (Ma et al., 2020b; Yan et al., 2020). Our work develops methods that enable the greater understanding of adsorption behavior of VOC on varying materials that have different affinities and kinetics. In addition, the methods used here can help to identify how different nanostructures can enhance adsorption or hydrophobicity when compared with identical smooth materials.

The usage of volatile chemical products (VCPs) accounts for a significant contribution of VOC emissions (Gligorovski and Abbatt, 2018). The increasing usage of VCPs has the potential to exacerbate VOC adsorption on REOs and other oxide surfaces owing to their higher reactivity with hydroxyl (OH) radicals. Our work shows that VOC adsorption on REO surfaces stems from the combined effects of chemisorption and physisorption. Thus, it is essential to further investigate how different types of VOCs adsorbed in different conditions promote or hinder VOC adsorption toward specific needs.

REOs are frequently used as catalysts for various energy-related applications ranging from catalytic combustion of fuel gas and biofuel synthesis to fuel cells and air purification. Hydrophobicity of a catalytic surface can enhance the catalytic efficiency of the oxidation and synthesis of organic compounds and performance of the proton-exchange membrane fuel cells or solid oxide fuel cell. Thus, understanding the wettability of REO surfaces and fabrication of chemically and thermally robust REO-based hydrophobic catalyst surfaces is crucial.

In this study, we investigated the mechanisms governing the wettability of REOs to address the long-term debate within the surface science community. Wettability characterization of REOs and control samples exposed to ambient laboratory conditions and to controlled hydrocarbon conditions after using a variety of surface cleaning methods resulted in an increase in contact angle over time that is directly correlated to the presence of hydrocarbons (surface coverage) adsorbed on the surface. Our results demonstrate that REO surfaces are intrinsically hydrophilic but become hydrophobic as they adsorb hydrocarbons. The adsorption of hydrocarbons from airborne VOCs and controlled hydrocarbon atmospheres was confirmed by multiple independent surface chemistry analysis methods, including XPS, AFM, and ToF-SIMS. ToF-SIMS experiments suggest that the adsorption mechanism on REOs is composed of both chemisorption and physisorption. The chemisorbed hydrocarbon promotes further physisorption owing to the higher affinity between similar hydrocarbon molecules. Our work offers a better fundamental understanding of the intrinsic wettability of REOs and provides key design guidelines for REO-based hydrophobic coatings and related catalytic materials.

## Limitations of the study

In this study, we attempted to minimize the adsorption of VOCs on the surfaces immediately after sample fabrication and cleaning prior to subsequent experimental procedures for characterization. These subsequent characterizations include apparent advancing and receding contact angle measurements as well as surface analyses. However, it is practically impossible to completely prevent the instantaneous adsorption of VOCs on a sample exposed to laboratory air. In our work, we assume that *in situ* experiments in UHV conditions for surface characterization provide a clearer surface chemistry distinction between surfaces that may have adsorbed VOCs and surfaces that were in pristine condition devoid of VOC adsorption.

## STAR★Methods

### Key resource table

**Table:** 

REAGENT or RESOURCE	SOURCE	IDENTIFIER
**Chemicals**		

Acetone	Sigma-Aldrich	67-64-1
Isopropyl Alcohol	Sigma-Aldrich	67-63-0
Hexane (Anhydrous, ≥95%)	Sigma-Aldrich	110-54-3
Heptane (Anhydrous, ≥99%)	Sigma-Aldrich	142-82-5
Octane (Anhydrous, ≥99%)	Sigma-Aldrich	111-65-9
Nonane (Anhydrous, ≥99%)	Sigma-Aldrich	111-84-2
Decane (Anhydrous, ≥99%)	Sigma-Aldrich	124-18-5

**Rare Earth Oxides**		
Cerium oxide	K. J. Lesker	EJTCEOX401A2
Erbium oxide	K. J. Lesker	EJTEROX401A2
Ytterbium oxide	K. J. Lesker	EJTYBOX401A2
Niobium oxide	K. J. Lesker	EJTNBOX401A2
Copper	K. J. Lesker	EJTCUXX401A2
Silicon oxide	K. J. Lesker	EJTSIO2451A2

**Other**		
Microgoniometer	Kyowa	MCA-3
X-ray photoelectron spectroscopy (XPS)	Kratos	Axis ULTRA
Time-of-flight secondary ion mass spectroscopy (ToF-SIMS)	PHI	Trift III
Sputter	AJA	Orion 3V
VOC meter	Omega	HHAQ-107

### Resource availability

#### Lead contact

Further information and requests for resources and reagent should be directed to and will be fulfilled by the lead contact, Nenad Miljkovic (nmiljkov@illinois.edu).

#### Materials availability

This study did not generate an unique material to be shared.

### Method details

#### Surface preparation and cleaning

##### Surface preparation

To fabricate REO samples, we used 1 inch diameter sputtering targets acquired from K.J. Lesker, USA. All sputter targets were fabricated at K.J. Lesker and sealed in an argon (Ar) environment to prevent contamination and oxidation (See Key Resource Table in STAR Method for a detailed specification of sputtering targets used). Briefly, cerium oxide (CeO_2_), erbium oxide (Er_2_O_3_), ytterbium oxide (Yb_2_O_3_), niobium oxide (Nb_2_O_5_), and copper (Cu) sputter material were procured. We chose sputtering targets as the test samples due to their well-known and controlled material purity. Ceramic sputtering targets typically display roughness (*r* > 5 μm) as well as residual porosity caused from the sintering procedure during manufacturing. To minimize the effect of surface roughness and porosity on the wettability on the test surfaces, 10 nm thin films of CeO_2_, Er_2_O_3_ and Yb_2_O_3_ REOs, and Nb_2_O_5_ as a transition metal oxide, were deposited onto test grade (1 0 0) Si wafers from University Wafer (United States). We used an AJA ORION 3 sputtering system (AJA International Inc., United States) and a ST20 ORION magnetron gun (AJA International Inc., United States), with radiofrequency (RF) plasma at a maximum power between 40-60 W. These settings achieved 0.03-0.07 Å/min deposition rate. During the process, the power ramped up and down at a rate of 20 W/min to prevent excessive thermal stress on the sputter targets.

##### Surface cleaning and preparation

We utilized a plasma cleaner (PDC-001, Harrick Plasma, USA) and air as a process gas for plasma cleaning. We also conducted organic solvent cleaning of the surfaces in an ultrasonic bath (CPX2800H, Branson) with acetone (C_3_H_6_O, 99% purity, Fisher Scientific), isopropyl alcohol (IPA, C_3_H_8_O, 99% purity, Fisher Scientific) and deionized (DI) water in series for 10 minutes each, followed by another IPA wash and drying with a stream of N_2_ in order to remove organic contaminants.

##### Surface wettability characterization

Water droplet contact angles on the surfaces were measured using a microgoniometer (MCA-3, Kyowa Interface Science, Japan). Both the apparent advancing (*θ*_a_) and apparent receding (*θ*_r_) contact angles were measured with deionized water droplets (≈25 μm in diameter) deposited using a contactless piezoelectric microdroplet injector at a rate of 50–90 droplets/sec (Günay et al., 2017). We measured both the advancing and receding contact angles to eliminate ambiguity commonly encountered in contact angle reporting. By doing so, we also quantify the contact angle hysteresis (*θ*_a_ - *θ*_r_) that is a key to understanding the wettability and adhesion (Cha et al., 2020; Ho et al., 2021; Li et al., 2021). Although past works report an ‘equilibrium’ contact angle, this is not rigorous as true equilibrium between the liquid droplet and surrounding vapor is difficult to attain or maintain. Therefore, the advancing and receding contact angles provide a better reflection of the surface wettability and adhesion than the equilibrium contact angle (Ma et al., 2020b). Contact angle data were analyzed by image processing using a software (FAMAS, interFAce Measurement & Analysis System) provided by the instrument manufacturer. To ensure repeatability and statistical significance, contact angles were measured on 10 separate location on each sample and averaged with reported standard deviation.

#### Surface chemistry characterization

##### X-ray photoelectron spectroscopy (XPS)

X-ray Photoelectron Spectroscopy (XPS) was performed to characterize surface chemistry. A Kratos Axis ULTRA instrument (Kratos Analytical, Ltd., UK) was used at grazing (15°) and normal takeoff angles. We analyzed XPS spectra using CasaXPS software (Casa Software, UK).

##### Time-of-flight secondary ion mass spectroscopy (ToF-SIMS)

Time-of-Flight Secondary Ion Mass Spectroscopy (ToF-SIMS) was performed to analyze the chemical species adsorbed on the surface in detail using a PHI Trift III system (PHI, Japan). We conducted surface analysis and depth profiling to examine how the surface chemistry changes over time. We used a gold (Au) primary liquid metal ion gun (LMIG) for both positive and negative secondary ion detection at bunched mode for surface analysis. A cesium ion (Cs^+^) gun was used for depth profiling for negative ions considering that the ions we expect to detect stem from REEs, oxygen, and carbon. The suppressor voltage and emission currents were 10 kV and 10 nA, respectively. The scanning area was 100 μm × 100 μm for surface analysis and 800 μm × 800 μm for depth profiling. The data was acquired and analyzed using WinCadence software (ULVAC-PHI, Japan).

##### Atomic force microscopy

We conducted tapping mode AFM to examine possible topological variation due to gas phase adsorption. We used an MFP-3D AFM (Asylum Research, United States) with a tapping mode AFM tip (AL300-G, BudgetSensors, United States), and a scan area of 2.5 μm × 2.5 μm. The AFM data was analyzed using IgorPro (Asylum Research, United States) and open-source software, Gwyddion (Nečas and Klapetek, 2012).

##### Air quality measurement

Indoor laboratory air quality was characterized using a commercialized VOC meter (HHAQ-107, Omega, USA) showing the presence of 20 ± 10 ppm of VOCs. The major sources of VOC in the laboratory indoor air can be solvent usage, vacuum pump oils, human breath and air circulation system (Gligorovski and Abbatt, 2018).

## Data Availability

• All data reported in this paper will be shared by the lead contact upon request. • This paper does not report any original code. • Any additional information required to reanalyze the data reported in this paper is available from the lead contact upon request.
