# Supplementary material for: The apparent surface free energy of rare earth oxides is governed by hydrocarbon adsorption
Source: iScience. 2021 Dec 25;25(1):103691. doi: 10.1016/j.isci.2021.103691 (PMC8752908; doi:10.1016/j.isci.2021.103691)
Supplement: Document S1. Figure S1 and Tables S1–S3 [file mmc1.pdf]

iScience, Volume *25*

Supplemental information

The apparent surface free energy of rare earth oxides is governed by hydrocarbon adsorption

Junho Oh, Daniel Orejon, Wooyoung Park, Hyeongyun Cha, Soumyadip Sett, Yukihiro Yokoyama, Vincent Thoreton, Yasuyuki Takata, and Nenad Miljkovic

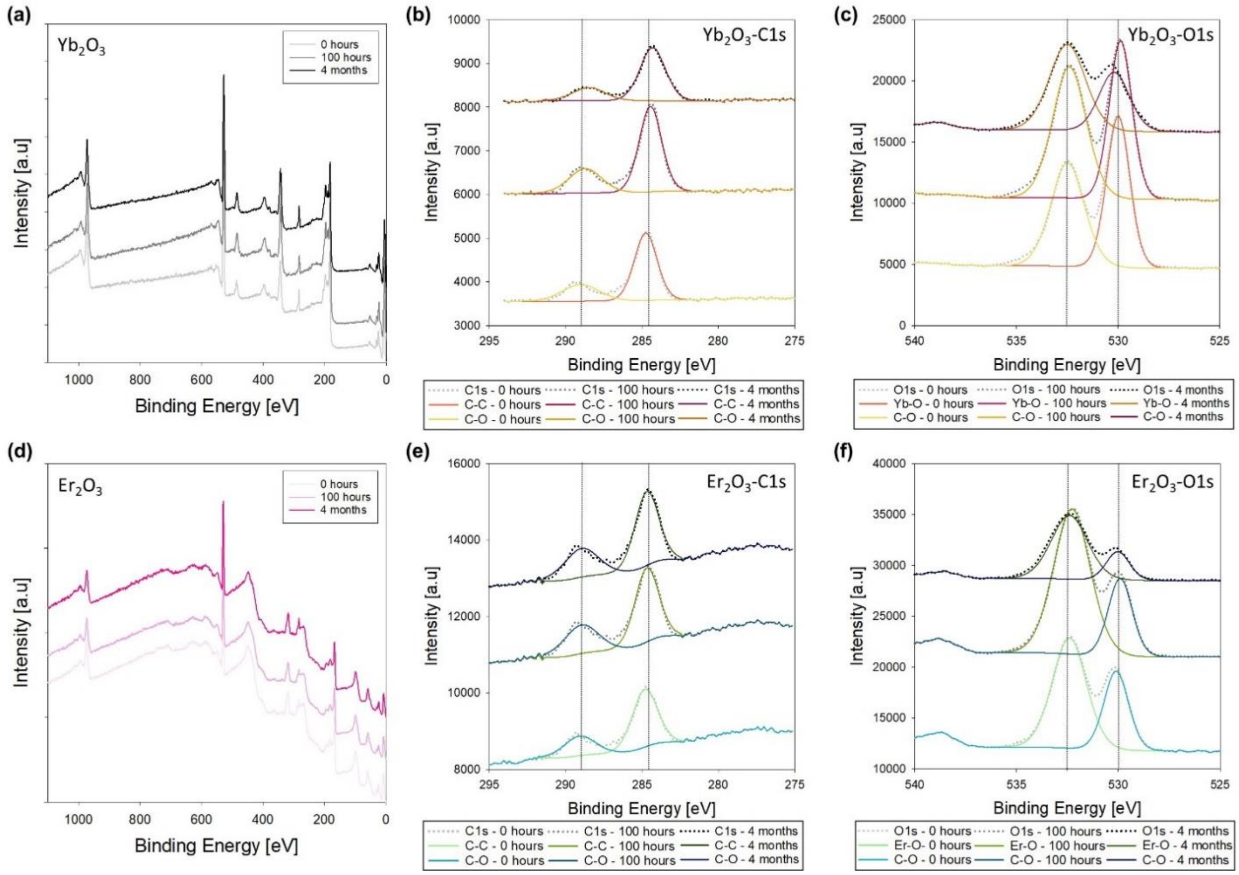

Figure S1. X-ray photoelectron spectroscopy (XPS) results of ytterbium oxide (Yb_2O_3) and erbium oxide (Er_2O_3) deposited by sputtering on a (1 0 0) test grade silicon wafer exposed to indoor laboratory air (Urbana, Illinois, USA) for 0 hours, 100 hours, and 4 months. (Related to the Air Quality Measurement in STAR Methods) (a) Broadband XPS spectra and high-resolution narrow band XPS spectra for (b) C1s (275-295 eV) and (c) O1s (525-540 eV) for the 10 nm Yb_2O_3 film. (d) Broadband XPS spectra and high-resolution narrow band XPS spectra for (e) C1s (275-295 eV) and (f) O1s (525-540 eV) for the 10 nm Er_2O_3 film. (Related to the Surface Characterization in STAR Methods)

Table S1. Detailed analysis on XPS data obtained from ytterbium oxide (Yb_2O_3) before and after exposure to indoor air for 4 weeks and 4 months. The entire spectral data was used to quantify the elemental composition on the surfaces, and high-resolution narrow band spectra (C1s: 275-290 eV, O1s: 525-540 eV) provided chemical bonding of certain elements. (Related to the Surface Characterization in STAR Methods)

**Ytterbium oxide (Yb_2O_3)**									
Condition	Before exposure			4 weeks exposure			4 months exposure		
Elements	Yb 4d	C 1s	O 1s	Yb 4d	C 1s	O 1s	Yb 4d	C 1s	O 1s
At. %	27.56	21.02	51.41	20.54	26.94	52.52	20.17	38.21	41.62
Detailed Peak analyses	C 1s	C-C<br>70.99	C-O<br>29.01	C 1s	C-C<br>76.55	C-O<br>23.45	C 1s	C-C<br>77.52	C-O<br>22.48
	O 1s	Yb-O<br>79.1	C-O<br>20.9	O 1s	Yb-O<br>71.77	C-O<br>28.23	O 1s	Yb-O<br>64.49	C-O<br>35.51

Table S2. Comparison of positive secondary ions detected from the ToF-SIMS analysis on the CeO_2 film deposited on a polished Si wafer that was exposed to a controlled atmosphere containing a range of different saturated hydrocarbons. The ion counts for each ion were normalized by CeO^+, which is one of dominating positive secondary ions derived from CeO_2. (Related to the Surface Characterization in STAR Methods)

Sample	C_6H_{14}	C_7H_{16}	C_8H_{18}	C_9H_{20}
**C_2H_3/CeO**	9.62%	7.40%	7.34%	5.54%
**C_2H_5/CeO**	11.49%	9.01%	8.27%	6.45%
**C_3H_3/CeO**	6.81%	6.20%	5.62%	4.34%
**C_3H_5/CeO**	18.25%	14.94%	13.45%	9.30%
**C_3H_7/CeO**	23.14%	18.26%	14.84%	10.75%
**C_4H_7/CeO**	16.94%	13.50%	10.47%	6.97%
**C_4H_9/CeO**	15.28%	13.44%	8.99%	6.55%
**C_5H9/CeO**	8.71%	6.75%	5.10%	3.19%
**C_5H_{11}/CeO**	5.95%	4.55%	2.99%	2.25%
**Ce/CeO**	**69.49%**	**47.88%**	**48.94%**	**42.69%**
**CeH/CeO**	36.87%	22.62%	21.78%	15.77%
**CeO_2/CeO**	4.40%	4.99%	4.93%	4.82%
**CeO_2H/CeO**	4.35%	4.13%	3.65%	3.18%
**CxHy/CeO**	**116.17%**	**94.04%**	**77.06%**	**55.35%**

Table S3. Comparison of negative secondary ions detected from ToF-SIMS analysis on a CeO_2 film deposited on a polished Si wafer after being exposed to a controlled atmosphere with different saturated hydrocarbons. The ion counts for each ion were normalized by O^-, which is one of the most dominant negative secondary ions derived from CeO_2. (Related to the Surface Characterization in STAR Methods)

Sample	C_6H_{14}	C_7H_{16}	C_8H_{18}	C_9H_{20}
**H/O**	235.87%	145.69%	141.70%	83.30%
**OH/O**	91.04%	76.29%	75.44%	65.55%
**OH_3/O**	36.37%	15.27%	25.73%	28.20%
**O_2H_3/O**	9.62%	8.43%	5.80%	11.94%
**O_2H_5/O**	4.16%	3.97%	3.32%	5.01%
**CNO/O**	4.55%	8.89%	5.26%	5.88%
**C_2HN_2O/O**	1.69%	1.34%	1.44%	1.30%
**C_3HO_4/O**	0.48%	0.60%	0.49%	0.93%
**$C_3H_3O_5$/O**	0.13%	0.14%	0.15%	0.12%
